# Quadriceps and hamstring anterior cruciate ligament reconstruction differ only marginally in function after the rehabilitation: a propensity score-matched case–control study

**DOI:** 10.1007/s00167-023-07422-y

**Published:** 2023-04-30

**Authors:** Daniel Niederer, Matthias Keller, Sarah Jakob, Wolf Petersen, Natalie Mengis, Lutz Vogt, Daniel Guenther, Georg Brandl, Björn H. Drews, Michael Behringer, David A. Groneberg, Thomas Stein

**Affiliations:** 1 Institute of Occupational, Social and Environmental Medicine, Goethe University Frankfurt, Frankfurt am Main, Germany; 2 OSINSTITUT Ortho and Sport, Munich, Germany; 3 Klinik für Orthopädie und Unfallchirurgie, Berlin, Germany; 4 Arcus Sportklinik, Pforzheim, Germany; 5 Department of Orthopaedic Surgery, Trauma Surgery, and Sports Medicine, Cologne Merheim Medical Center, Witten/Herdecke University, Witten, Germany; 6 Department of Orthopaedic Surgery II, Herz-Jesu Krankenhaus, Vienna, Austria; 7 St. Vinzenz Clinic Allgäu, Pfronten, Germany; 8 SPORTHOLOGICUM Frankfurt—Center for Sport and Joint Injuries, Frankfurt am Main, Germany; 9 Department of Sports Medicine and Exercise Physiology, Goethe University Frankfurt, Frankfurt am Main, Germany; 10 KSA Aarau/Spital Zofingen, Aarau, Switzerland; 11 University Hospital, Basel, Switzerland

**Keywords:** Propensity score, Autograft, Functional capacity, Return to sports

## Abstract

**Purpose:**

To determine potential quadriceps versus hamstring tendon autograft differences in neuromuscular function and return to sport (RTS)-success in participants after an anterior cruciate ligament (ACL) reconstruction.

**Methods:**

Case–control study on 25 participants operated on with an arthroscopically assisted, anatomic ipsilateral quadriceps femoris tendon graft and two control groups of 25 participants each, operated on with a semitendinosus tendon or semitendinosus-gracilis (hamstring) tendon graft ACL reconstruction. Participants of the two control groups were propensity score matched to the case group based on sex, age, Tegner activity scale and either the total volume of rehabilitation since reconstruction (*n* = 25) or the time since reconstruction (*n* = 25). At the end of the rehabilitation (averagely 8 months post-reconstruction), self-reported knee function (KOOS sum scores), fear of loading the reconstructed knee during a sporting activity (RSI-ACL questionnaire), and fear of movement (Tampa scale of kinesiophobia) were followed by hop and jump tests. Front hops for distance (jumping distance as the outcome) were followed by Drop jumps (normalised knee joint separation distance), and concluded by qualitative ratings of the Balanced front and side hops. Between-group comparisons were undertaken using 95% confidence intervals comparisons, effect sizes were calculated.

**Results:**

The quadriceps case group (always compared with the rehabilitation-matched hamstring graft controls first and versus time-matched hamstring graft controls second) had non-significant and only marginal higher self-reported issues during sporting activities: Cohen’s *d* = 0.42, *d* = 0.44, lower confidence for RTS (*d* = − 0.30, *d* = − 0.16), and less kinesiophobia (*d* =  − 0.25, *d* = 0.32). Small and once more non-significant effect sizes point towards lower values in the quadriceps graft groups in the Front hop for distance limb symmetry values in comparison to the two hamstring control groups (*d* =  − 0.24, *d* = − 0.35). The normalised knee joint separation distance were non-significantly and small effect sized higher in the quadriceps than in the hamstring groups (*d* = 0.31, *d* = 0.28).

**Conclusion:**

Only non-significant and marginal between-graft differences in the functional outcomes at the end of the rehabilitation occurred. The selection of either a hamstring or a quadriceps graft type cannot be recommended based on the results. The decision must be undertaken individually.

**Level of evidence:**

III.

**Supplementary Information:**

The online version contains supplementary material available at 10.1007/s00167-023-07422-y.

## Introduction

After an anterior cruciate ligament (ACL) rupture and reconstruction, neuromuscular performance, mostly rated by jump- or hop-performances and movement quality, are predictive for a subsequent second ACL injury [2, 11, 13]. Consequently, knowledge on neuromuscular performance is crucial when reconstruction-, rehabilitation- and return to sport-success is rated after an ACL reconstruction.

Semitendinosus (alone or in combination with gracilis; = hamstring) and quadriceps femoris tendon grafts show comparable graft survival rates, clinical stability and functional outcomes when compared to each other [14, 15]. In contrast functional outcomes, such as knee extensor muscle strength deficiency, might be superior in knees which were reconstructed with a hamstring graft than in those with a quadriceps graft [14, 22]. Although numerous randomised controlled and observational studies have compared quadriceps and hamstring grafts, little is known on potential differences between hamstring and quadriceps graft-based reconstructions on return to sport success rates.

In most studies on this topic, the comparative functional measurements were taken at pre-selected or randomly selected time points. As the duration until rehabilitation completion and return to sports success is highly variable between individuals [24], a comparison at the real end of the individual rehabilitation [27], and not at a hypothetical end or at a random point in time, might be more promising when the functional outcomes of a graft type selection should be compared. A comparison of individuals who were reconstructed with a quadriceps tendon graft to participants with both, a rehabilitation-volume matched and a time-matched hamstring graft, may be the most valid approach to compare functional outcomes and return to sport success between different grafts.

Therefore, the purpose of the present study was to compare self-reported and objective functional outcomes, as well as return to sport success, from participants after a quadriceps autograft reconstruction with both a rehabilitation volume-matched and a time-matched hamstring autograft group. We hypothesised that (1) the quadriceps group shows inferior self-report and (2) objective functional values than the hamstring autograft groups and that (3) no between-group differences in return to sport success rates between the groups occurs.

## Materials and methods

This propensity score matched case–control study was conducted as a part of the PReP project [17]. The independent Ethics Committee of the Hessen Regional Medical Council Ethical (reference approval no. FF 104/2017) and, subsequently, each centre’s responsible ethics committee provided approval. Each participant signed informed written consent for participating in the intervention study prior to enrolment.

The study was planned and performed in agreement with the Declaration of Helsinki (Version Fortaleza 2013). The study was pre-registered in the German Clinical Trials Register (DRKS), 01. October 2018, DRKS00015313).

### Participants

Individuals with an acute unilateral ACL rupture and having passed an arthroscopically assisted, anatomic ipsilateral quadriceps femoris or a hamstring tendon graft ACL reconstruction were included. Secondary inclusion criteria were (1) being < 36 years of age, (2) have been engaged in any type of sport prior to the ACL injury and (3) aimed to return to their previous sporting activity.

Exclusion criteria consisted of a meniscus lesion with a diameter of > 2 cm, (2) a cartilage lesion > ICRS II°, previous musculoskeletal surgery of the uninvolved (contralateral) leg, leg malalignment > 5°, a multi-ligament injury pattern, severe post-operative complications such as graft failure, arthrofibrosis, or a subsequent second knee injury, acute or chronic inflammation of the musculoskeletal system or muscle soreness and pregnancy.

### Autograft selection

The autograft was selected based on biomechanical and individual criteria. When it was, from the athlete point of view of the participant and/or due to the type of activity, important to preserve the hamstring complex and, thus, to avoid damage to the knee flexor compartment [22], a quadriceps graft was usually selected. From a biomechanical point of view, small hamstring tendon sizes may not be suitable for a graft in certain patients [21].

### Matching

To match two counterpart to each quadriceps graft participant, we screened all 229 participants with a hamstring graft and a measurement at the end of the individual rehabilitation included in the PReP-cohort [17]. A logistic regression propensity score, utilising a matching ratio of 1:1, was subsequently performed. For each participant in the quadriceps group, the matching procedure was performed twice. Once with the total volume of rehabilitation measures since reconstruction and once with the time since reconstruction as one of the matching variables. In both matching groups, the other matching variables were: sex, age and the pre-injury Tegner activity scale. As a result of this procedure, the two resulting comparator groups contained duplicate persons and should, therefore, not be compared to each other but only to the quadriceps group.

### Sample

The quadriceps graft (case) sample consisted of *n* = 25 (12 females and 13 males, 25.8 ± 5.7 years) and, the hamstring graft (control) sample of *n* = 35 (17 females and 18 males, 24.4 ± 4.1 years) individual participants. *N* = 10 participants were included in both of the hamstring graft control groups.

### Individual rehabilitation and measurement time point determination

To ensure that they were not only called just once at the hypothetical end of rehabilitation, all participants were prospectively monitored until their individual rehabilitation completion through a series of telephone calls [5]. The prescription of the individual medically prescribed rehabilitation followed a stepwise function-based periodisation and progression [4, 24, 26]. Restoring the knee range of motion was followed by a function-based progression to strengthening and neuromuscular motor control training. The decisions on rehabilitation completion were made based on the formal completion of the medically prescribed rehabilitation measures, the integration of the available evidence and guidelines, the stakeholders’ clinical experience and the athlete's own preferences [1]. More detailed, full knee range of motion compared with the uninjured side, minimal effusion present (trace or less), ability to hop on one leg without pain and participating in a running progression programme, and psychological readiness for RTS were the release criteria [27]. In addition, the participants had to be cleared for their sport-specific training components and for hopping on one leg by their treating orthopaedic surgeon and physical therapists [5]. Due to local and health-assurance differences, minor between-participant differences regarding the exact design and structuring of the rehabilitation measures may exist.

### Measurements and outcomes

In the structured telephone interview at inclusion (at 1–3 weeks after the reconstruction), the participants reported socio-demographic and anthropometric details, injury mechanisms (contact free, indirect contact, direct contact), all (rehabilitation) measures taken between injury and reconstruction [8] and the pre-injury type(s) of sport and volume of training (recreational/low-level competitive, semi-professional [16], professional). Numerous surgery-specific outcomes were retrieved from the surgery report: autograft type (quadriceps femoris, semitendinosus, or semitendinosus-gracilis), tendon folding (from three times up to eight times) and the tendon diameter [mm].

The outcome assessments took place at the individually determined end of the rehabilitation and consisted of a battery of questionnaires, followed by a series of hop and jump tests. Knee function and symptoms were reported using the Knee injury and osteoarthritis outcome score (KOOS) subscales: sport (SPORT), pain (PAIN), symptoms (SYMPTOMS) and activities of all daily living (ADL). The KOOS thresholds/cut-offs for non-restricted ratings (“non-symptomatic”) were: PAIN 89 points, SYMPTOMS 83, ADL 95 and SPORT 72 points [18]. To report the potential fear of loading their reconstructed knee during a sporting activity, the participants completed the RTS after ACL injury questionnaire (RSI-ACL). For the ACL-RSI (11-point Likert scale), a total score value of > 56 indicates sufficient confidence for RTS [10]. Other self-reported outcomes were the Tegner activity scale to measure the participant’s sporting activity level and RTS and the Tampa scale of kinesiophobia (TSK), to measure their fear of movement. The volume, type, intensity and duration of all rehabilitation/sport/exercise measures undertaken since reconstruction were further assessed. When the same type and level of sport as that prior to the injury, irrespective of competition or training, was reached, the participant was treated as “RTS-successful” (yes/no at the time of the measurements). All these self-reported outcomes were collected in an online-survey on www.surveymonkey.de.

After a specific warm-up (jumping jacks and steps), the Front hop for distance (i.e. single leg hop for distance) was performed. The cut-off/threshold for the present case was selected to be an LSI of 90% for the Front hop for distance. The test conduction and measurement quality criteria can be found in the supplemental file 1.

The Drop jump screening test followed. A normalised knee separation distance at the jump’s reversal point of 60% was selected as the cut-off threshold for the following ratings and analyses [20]. The detailed test conduction and measurement quality criteria can be found in the supplemental file 1.

For the sagittal plane landing quality rating, the Balance front hop test [12] was performed. The frontal plane landing quality rating was undertaken using the Balance side hop test [12]. The test conduction and measurement quality criteria can be found in the Supplemental File 1.

All hop/jump tests were performed by self-administration and filmed from a frontal position (3 m distance). The participants used their own smartphone cameras. The videos were expert-rated using the investigator-blinded videos. Participants were educated on how to perform the jumps and hops. In cases of incorrect execution, the tests and, where needed, the instructions for the tests were repeated. This is valid when compared to 3D motion-capture systems for the analyses of sagittal plane knee angles [7].

### Statistical analysis

The required sample size was estimated based on the effects for the self-reported sport-associated function (KOOS-SPORT) at a 1-year post-surgery follow-up [14]. The quadriceps group reached a mean value of 70 (standard deviation 23) points and the hamstring graft group of 76 (16) points. Adapted to a matched pairs-design (paired tests) with a suggested between matching-pairs correlation coefficient of 0.7 and the calculated Cohen’s *dz* of 0.36; at least 25 full datasets per group are needed to be included in the analyses if a significant result should be found under an 5% alpha and a 20% beta error probability. Thus, 25 participants with a quadriceps graft had to be recruited and 2 × 25 hamstring graft participants from the PReP cohort had to be included for matching purposes.

The alpha-error threshold was set at 5% for all inference statistical analyses The analyses were performed with SPSS (Version 28, IBM SPSS, USA).

Main between-graft comparisons for any interval scaled or pseudo-interval scaled outcomes were undertaken based on the means and 95% confidence intervals comparisons plus between group effect size comparisons. The confidence intervals were displayed separated by groups, while comparisons were undertaken using confidence intervals overlapping comparisons and by Cohen’s *d* comparisons between groups (quadriceps in comparisons to one of the hamstring groups at each time).

The numbers (and %) of the participants fulfilling the pre-defined and literature-based cut-offs, again separated by group, were displayed as absolute numbers. Inference-statistically, associations of RTS success and fulfilling the functional cut-offs were checked using Chi^2^-tests or the Fisher's exact test, depending on the structure. The groups were compared using relative risks (with 95% confidence intervals).

Explorative secondary analyses contained linear mixed models for repeated measures. The time effects were modelled as random effects (and factors), the other independent variables (group) and covariates (potential confounders and effect modifiers) as fixed effects.

## Results

No participant was excluded, no one withdrew his/her consent for participation. No adverse events or serious adverse events occurred. All sport-, injury- and surgery-specific characteristics of the study sample can be found in Table 1.

**Table 1:** Numeric and percentage distributions of all baselines and traits: sport-, injury- and surgery-specific characteristics of the study sample

Domain	Outcome	Value/unit	Hamstring rehabilitation-matched		Quadriceps-graft		Hamstring time-matched	
			Number	%	Number	%	Number	%
Sport	Athletic level	Recreational/low-level competitive	23	92	20	80	21	84
		Semi-professional or professional	2	8	5	20	4	16
	Tegner activity level pre-injury^a^	3	2	8	2	8	4	16
		4	4	16	4	16	5	20
		5	1	4	1	4	2	8
		6	5	20	5	20	3	12
		7	9	36	9	36	6	24
		8	1	4	1	4	1	4
		9	3	12	3	12	3	12
		10	0	0	0	0	1	4
Injury	Injury mechanism	Contact free	18	72	13	52	15	60
		Indirect contact	5	20	6	24	6	24
		Direct contact	2	8	6	24	3	16
Surgery	Graft type (tendon)	Semitendinosus	12	48	0	0	13	52
		Semitendinosus-gracilis	13	52	0	0	12	48
		Quadriceps femoris	0	0	25	100	0	0
Covid-19		Pre- or post-restriction	7	28	15	60	5	20
		During restriction	18	72	10	40	20	80

Domain	Outcome	Unit	Mean	SD	Mean	SD	Mean	SD
Demographic	Body mass index	kg/m^2^	24.6	4.3	23.4	3.4	23.3	4.3
Sport	Training volume pre-injury	units/week	3.3	1.2	4	1.7	3.3	1.7
		min/unit	105	69	95	30	91	21
Surgery	Time between injury and reconstruction	days	86 (median)	74	89 (median 56)	79	80 (median 49)	74

The two control groups contained 10 duplicates each

*SD* Standard deviation

^a^Matching variable

The hamstring rehabilitation-matched group showed a significantly shorter time period since reconstruction than the quadriceps graft group (Additional Fig. 1A in the Supplement). In this period, the total volume of exercise (rehabilitation measures and sport) in minutes was significantly larger in the time-matched hamstring participants than in the quadriceps graft group individuals (Additional Fig. 1A in the Supplement).

**Fig. 1:**
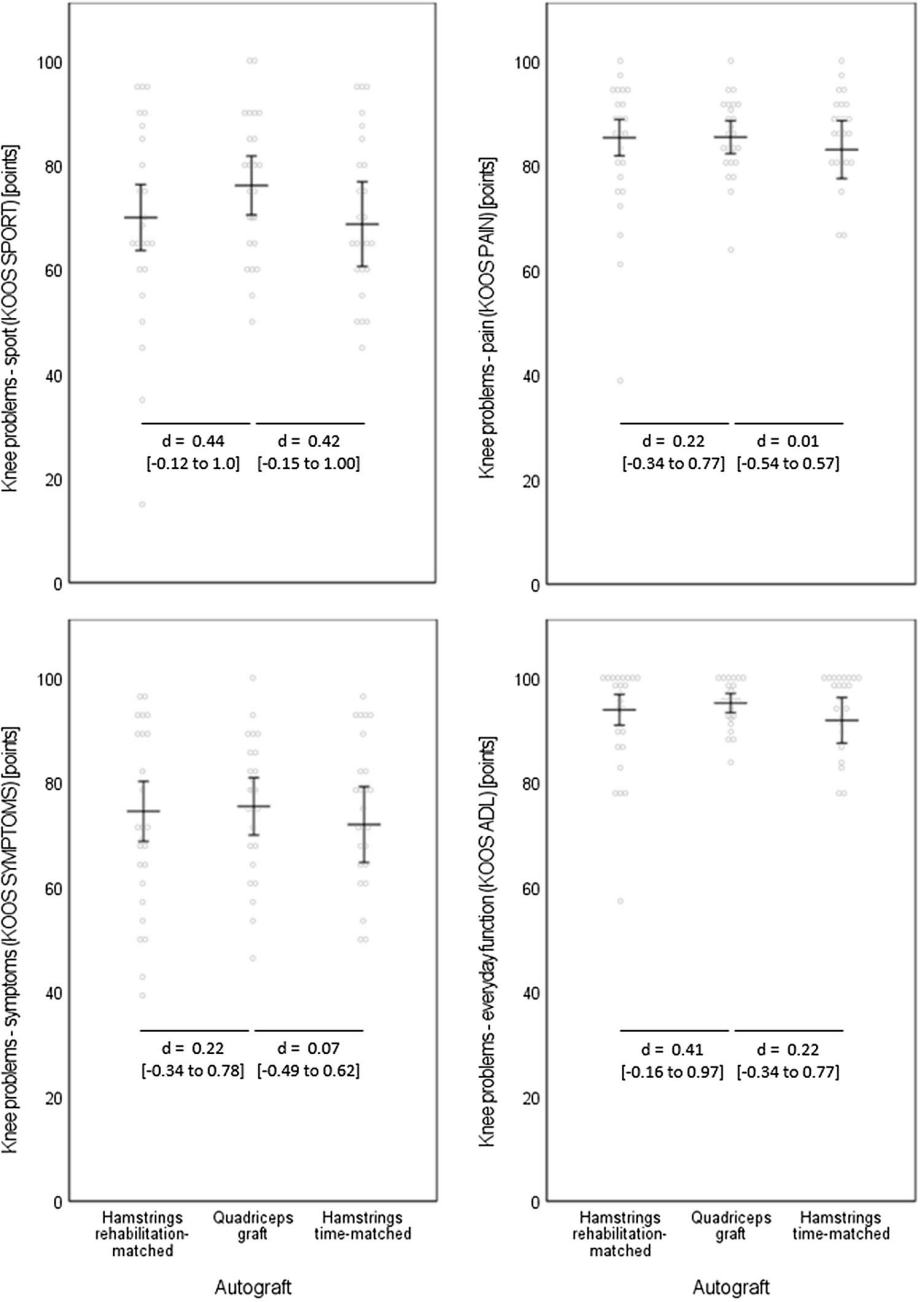
Self-reported knee problems. Data are displayed as individual values (dots) and as group means (horizontal lines) with 95%-confidence intervals (vertical lines). Effect sizes are displayed as Cohen’s *d* (*d*) with [95% confidence intervals]. *KOOS* The Knee Injury and Osteoarthritis Outcome Score, *ADL* activities of daily living

Figure 1 displays the outcomes for the self-reported knee problems, as assessed by the KOOS questionnaire. Only marginal effect sized and non-significant differences between the quadriceps graft participants and either of the two hamstring matching groups occurred. In the self-reported problems during sporting activities (KOOS SPORT), the quadriceps graft group showed non-significantly higher values than the two hamstring control groups.

**Fig. 2:**
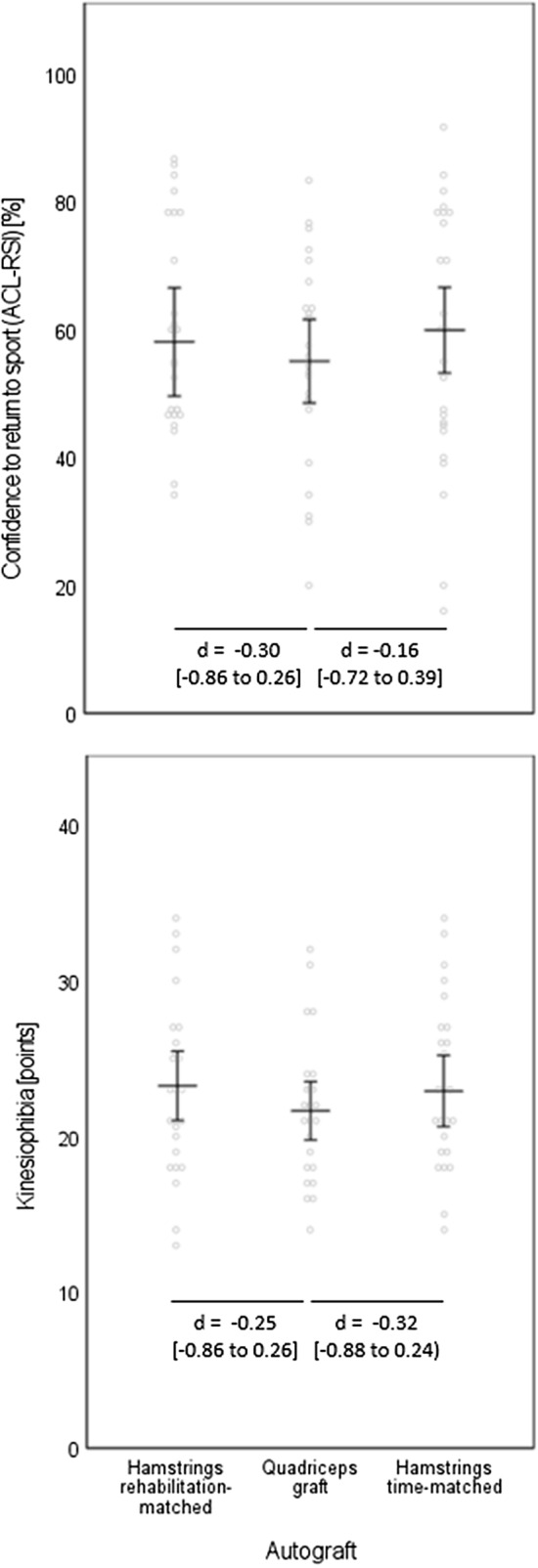
Self-reported knee confidence to return to sport and kinesiophobia. Data are displayed as individual values (dots) and as group means (horizontal lines) with 95%-confidence intervals (vertical lines). Effect sizes are displayed as Cohen’s *d* (*d*) with [95% confidence intervals]. *ACL-RSI* anterior cruciate ligament return to sport after injury questionnaire

The between-graft comparison for the confidence to return to sport (ACL-RSI) and for kinesiophobia revealed no between-graft differences (Fig. 2). Small effect sizes hinting towards less (although, again, non-significant) confidence for return to sport and towards less kinesiophobia in the quadriceps graft than in the two hamstring graft groups occurred.

**Fig. 3:**
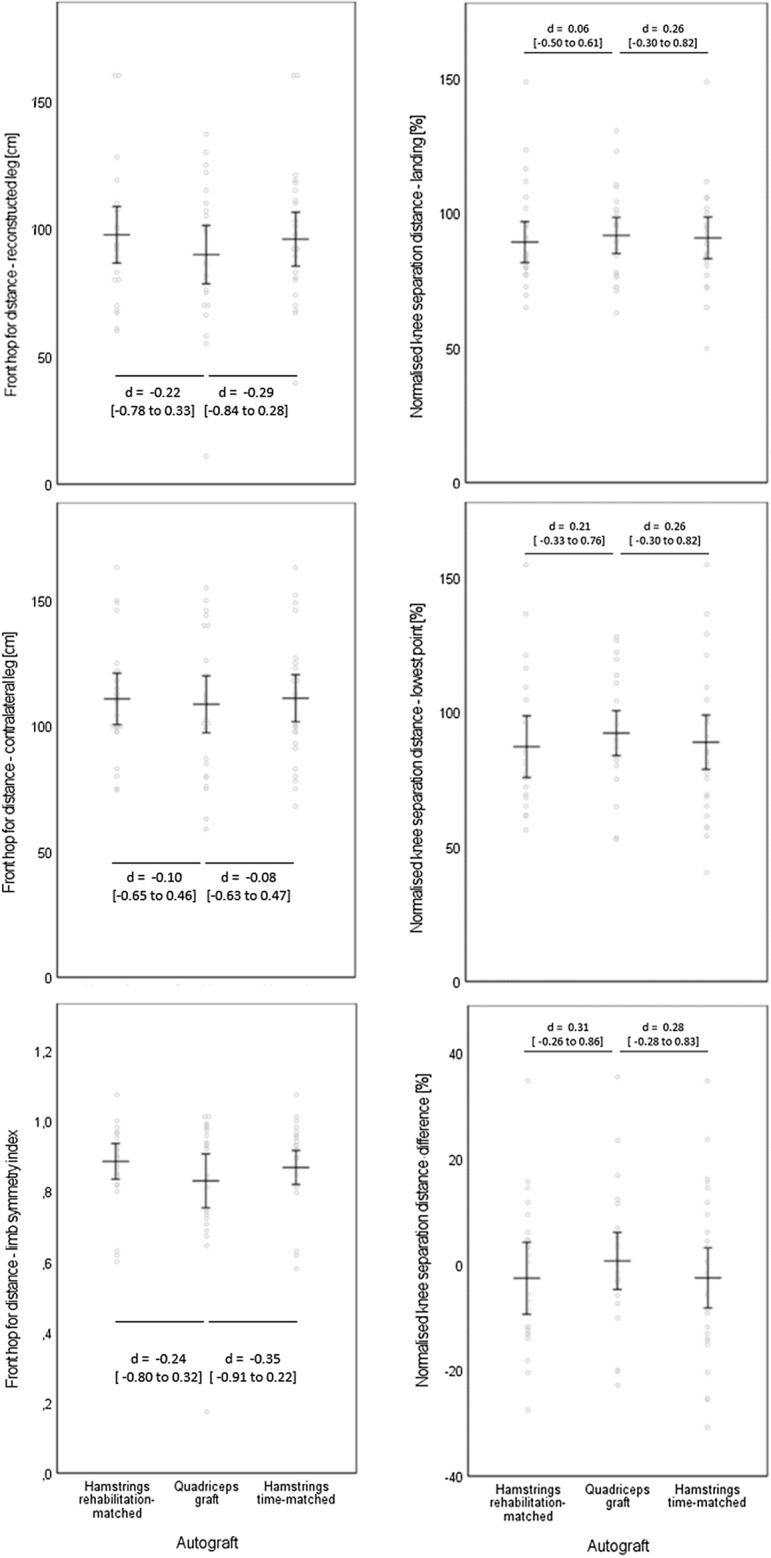
Results for the Front hop for distance (left part) and the Drop jump (as measured by the normalised knee separation distance, right), separated for the reconstructed (above) and contralateral (mid) leg, and for the limb symmetry index (below). Data are displayed as individual values (dots) and as group means (horizontal lines) with 95% confidence intervals (vertical lines). Effect sizes are displayed as Cohen’s *d* (*d*) with [95% confidence intervals]

In Fig. 3, the outcomes and between-graft comparisons for the Front hop for distance and for the Drop jump are displayed. No significant differences and only marginal to low effect sizes (favouring the two hamstring graft types in the Front hop and quadriceps graft participants in the Drop jump) were reached.

**Fig. 4:**
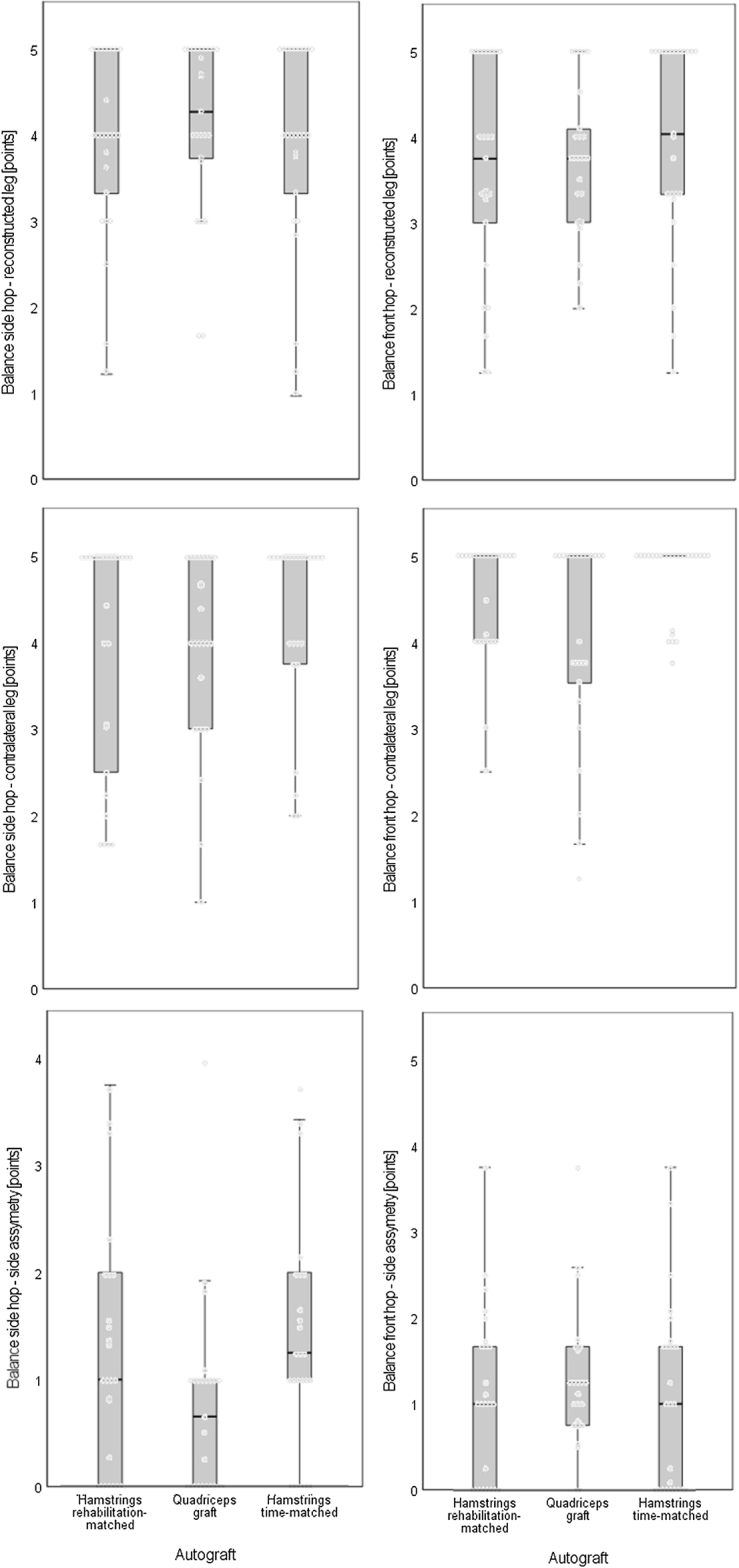
Results for the Balance hop tests, separated for the reconstructed (above) and contralateral (mid) leg, and for the limb symmetry index (below). Both the Balance side (left) and front hop (right) data are displayed as individual values (dots) and as boxplots with whisker bars, medians, and interquartile ranges

Only marginal and non-significant differences between the quadriceps graft participants and either of the two hamstring matching groups occurred in the two balance hopping performances (Fig. 4). The hamstring participants showed marginal and not significantly larger between-leg differences in the Balance side hop and better values in the Balance front hop performance with the contralateral leg.

Only 16% in the quadriceps graft group returned to their pre-injury type and level of sport. In the hamstring graft groups, this share was (rehabilitation-matched group) 32% and (time-matched control) 38%). The underlying absolute values for this percentage share and the relative risk including its confidence intervals are, likewise, for all other self-reported and objectively assessed functional outcomes, displayed in the Table 2, likewise.

**Table 2:** Frequency distribution of return to sport successes and all functional outcomes of which a validated cut-off -value are known. For each outcome, the absolute frequency distribution separated by each group and the corresponding relative risk (always the quadriceps graft cases in comparison to each of the hamstring graft comparator groups), including the confidence intervals of the relative risks, are displayed

Outcome	Coding	Hamstring rehabilitation-matched	Quadriceps graft			Hamstring time-matched
Return to the same type and level of sport	No [*n*]	15	21			17
	Yes [*n*]	9	4			8
	Relative risk [95% CI]	1.3 [0.94 to 1.92]		1.2 [0.90 to 1.7]		
Knee problems—sport (KOOS SPORT)	< 72 points [*n*]	15	10			15
	≥ 72 points [*n*]	10	15			10
	Relative risk [95% CI]	0.67 [0.37 to 1.2]		0.67 [0.37 to 1.2]		
Knee problems—pain (KOOS PAIN)	< 89 points [*n*]	14	15			14
	≥ 89 points [*n*]	11	10			11
	Relative risk [95% CI]	1.1 [0.67 to 1.72]		1.1 [0.67 to 1.72]		
Knee problems—symptoms (KOOS SYMPTOMS)	< 83 points [*n*]	19	18			17
	≥ 83 points [*n*]	6	7			8
	Relative risk [95% CI]	0.95 [0.68 to 1.32]		1.1 [0.74 to 1.5]		
Knee problems—everyday function (KOOS ADL)	< 95 points [*n*]	0	0			0
	≥ 95 points [*n*]	25	25			25
	Relative risk [95% CI]	1		1		
Confidence to return to sport (ACL-RSI)	< 56 points [*n*]	12	12			12
	≥ 56 points [*n*]	13	13			13
	Relative risk [95% CI]	1		1		
Front hop for distance limb symmetry index	< 90% [*n*]	11	13			14
	≥ 90% [*n*]	14	12			11
	Relative risk [95% CI]	1.2 [0.66 to 2.11]		0.93 [0.56 to 1.6]		
Normalised knee separation distance at lowest point	< 60% [*n*]	4	2			1
	≥ 60% [*n*]	21	23			24
	Relative risk [95% CI]	0.5 [0.10 to 2.5]		1.20 [0.19 to 20.6]		
Balance side hop—ACL leg	< 5 points [*n*]	16	12			16
	5 points [*n*]	9	13			9
	Relative risk [95% CI]	0.8 [0.45 to 1.2]		0.8 [0.45 to 1.2]		
Balance side hop Contralateral leg	< 5 points [*n*]	11	14			11
	5 points [*n*]	14	11			14
	Relative risk [95% CI]	1.3 [0.73 to 2.2]		1.3 [0.73 to 2.2]		
Balance front hop—ACL leg	< 5 points [*n*]	12	18			17
	5 points [*n*]	13	7			8
	Relative risk [95% CI]	1.5 [0.93 to 2.4]		1.1 [0.74 to 1.5]		
Balance front hop Contralateral leg	< 5 points [*n*]	4	12			8
	5 points [*n*]	21	13			17
	Relative risk [95% CI]	3.0 [1.3 to 18.3] *		1.5 [0.74 to 3.0]		

*n* number, *KOOS* Knee Injury and Osteoarthritis Outcome Score, *ACL-RSI* return to sport after injury, *ADL* activities of daily living, *CI* confidence interval

None of the outcomes was non-equally distributed between groups with the exception of the Balance front hop for the contralateral leg. Here, more participants of the quadriceps graft group than in the hamstring graft time-matched group had not reached the requested 5 points.

The results of the explorative linear mixed models can be found in the Supplemental Table 1. The graft type itself had a significant effect on knee problems during everyday functions; here, the quadriceps patients reported worse values (Supplemental Table 1). Furthermore, many co-variates such as age, time since reconstruction, having been rehabilitated during the COVID-associated restrictions, and the Tegner activity scale had a significant effect on the outcomes. In contrast, only minor non-significant interaction effects (co-variate*graft type) occurred: graft type* sex had a negative impact on kinesiophobia, whereas graft type*lockdown led to a decrease in both kinesiophobia and in the Front hop for distance limb symmetry index, likewise. Graft type*age significantly increased the normalised knee separation distance at landing.

## Discussion

The most important finding of the present study was the marginal differences in self-reported and objectively assessed function between participants with a quadriceps autograft reconstruction and those with hamstring autograft reconstructions. These marginal differences were independent of whether the hamstring control group was matched to the quadriceps graft case group in terms of rehabilitation amounts or in terms of time since reconstruction. Therefore, the hypotheses (1) and (2) must be rejected, while hypothesis (3) could be verified.

The participants completed their individual rehabilitation averagely at 8 months post-reconstruction. This value corresponds to reference studies in which the return to sport clearance in young, non-professional athletes was reached at 8.2 months post-surgery [23]. Thus, external validity of the sample seems to be given. The findings are consequently, generalizable to a young and physically active population following an isolated ACL tear and subsequent hamstring or quadriceps tendon graft reconstruction.

The non-significant and, according to the effects sizes, marginal differences between the quadriceps and hamstring-tendon grafts are in accordance with the current literature in the field [3, 22]. Neither in the self-reported functional ratings, such as the IKDC [3, 22], Tegner-, or Lysholm-score [22], nor in the objective functional outcomes, such as the Front hop for distance limb symmetry index [22], were between-graft type-differences frequent. Whilst the same picture is eminent for the knee flexor strength and hamstring to quadriceps strength ratios, the hamstring tendon autografts were associated with a better limb symmetry index in the knee extensor strength [22]. This may be a reason for the potential differences found in the present sample: in contrast to the Drop jump and Balance side hop performances, the Front hop for distance and the Balance front hop both need a sagittal plane deceleration with a major contribution from the knee extensors. The Drop jump, and, in particular, the Balance side hop do not need such a major contribution of the knee extensors. One must keep in mind that the results were non-significant and so the graft type discussion in this paper should be considered as explorative.

Sport-associated self-reported outcomes scores were, additionally, only slightly superior in the hamstring-graft groups, whereas the kinesiophobia values tended to be lower in the quadriceps group. An explanation for the latter finding may be found in the explorative analyses; the graft types may have acted differently during a lockdown in terms of kinesiophobia [19]. A smaller share of the quadriceps group than in the two hamstring graft groups performed their rehabilitation measures during restriction. Furthermore, as the COVID-associated restriction impacted on many psychosocial issues [6], kinesiophobia may also have been affected.

In general, upon individual rehabilitation completion, those participants with a rehabilitation-matched and those with a time-matched hamstring graft volume were not different than participants with a quadriceps tendon graft.

As only marginal between-graft differences in functional outcomes at the end of the rehabilitation occurred, the decision to selection of a hamstring or a quadriceps graft type cannot be undertaken based on the results. Beyond comparable functional findings of other studies, this conclusion is supported by findings of no differences in stability outcomes and in the odds for a Lachman test grade 0 differences between grafts at an early post-reconstruction time point [3, 22]. In contrast, the quadriceps autograft knees display significantly lower rates of donor site morbidity than the hamstring grafts, but higher knee extensor strength values [22]. It cannot, to sum up, be conclusively be recommended to use any of these two autografts based on the current literature. The decision must be undertaken individually. A small hamstring tendon size leads to the decision not to select a hamstring tendon as an autograft [22]. The need to avoid donor-site damages of the knee flexor, in, for example, in judokas and cyclists and of the knee extensors, in, for example, in hockey players, automatically excludes the respective tendons as a graft.

As only considerably low shares of the participants fulfilled all the functional cut-offs for a successful function at the end of their formal rehabilitation, the end of the rehabilitation is, thus, not the actual end of the rehabilitation [18]. One may conclude that either adding more sport-specific training or continuing rehabilitation exercises after the end of the medically prescribed formal rehabilitation measures may be required for recovery completion.

Future studies are warranted to determine potential differences in the trainability between the different graft types, both during rehabilitation itself at the early and late-stage, and during subsequent recurrence-preventive measures. A graft comparison in other samples such as adolescents or in older participants than ours would furthermore expand the transferability of the results.

From a pragmatic point of view and due to the research gap sketched above (the need for a rehabilitation- versus time-matching), a randomised controlled approach could not be adopted in the present study. Although meta-analyses found no significant differences in the effect estimates between the randomised controlled trials and observational studies [3], it still must be considered as a limitation.

In this study, only tests that were self-administered by the patient in view of the pandemic situation and the otherwise higher dropout rate were adopted; this approach was assumed to be the most practicable way. However, this setting may display a more vulnerable validity than laboratory measurements.

Although a state-of-the-art effect estimator was used, the LSI may overestimate one’s functional ability by masking bilateral deficits [9]. Recently, estimated pre-injury capacity levels (EPIC), instead of LSIs, were proposed to be more sensitive in predicting a second ACL injury [25].

By including 3 × 25 participants, the sample size calculation was followed; the analyses are, thus, considered as adequately powered. However, a larger sample size would have led to even more robust findings, although the clinical relevance of the (rather marginal and small effect [sizes]) still would question the clinical relevance of the between-graft differences.

## Conclusions

Only marginal and non-significant differences in functional outcomes between quadriceps and hamstring autograft reconstructions occur after anterior cruciate ligament ruptures at the end of rehabilitation. The decision for selecting a hamstring tendon or a quadriceps tendon autograft cannot conclusively be recommended based on these functional outcomes but must be undertaken individually.

## Supplementary Information

Below is the link to the electronic supplementary material.

Supplementary file1 (DOCX 96 kb)
