# Supplementary material for: Quadriceps and hamstring anterior cruciate ligament reconstruction differ only marginally in function after the rehabilitation: a propensity score-matched case–control study
Source: Knee Surg Sports Traumatol Arthrosc. 2023 Apr 30;31(8):3441–53. doi: 10.1007/s00167-023-07422-y (PMC10149044; doi:10.1007/s00167-023-07422-y)
Supplement: Supplementary file 1 — Supplementary file1 (DOCX 96 kb) [file 167_2023_7422_MOESM1_ESM.docx]

SUPPLEMENTAL FILES

Quadriceps and hamstring anterior cruciate ligament autografts differ only marginal in function after anterior cruciate ligament ruptures reconstruction differ only marginally in function after at the end of rehabilitation: a propensity score-matched case-control study

**1 Hop and jump test conduction and quality criteria**

First, the Front hop for distance (i.e., single leg hop for distance) was performed. The participants hopped frontally as far as possible with the test leg and had to land in a controlled manner. The hopping distance was measured toe to toe (at take-off and landing). The hands did not have to be kept on the hips, but could be used for hop and landing control. Three successful hops per leg (randomised order) were performed, with each leg’s best trials [cm] being selected for further analysis. The cut-off/threshold for the present case was selected to be an LSI of 90% for the Front hop for distance. The measurement properties for the Front hop for distance are excellent; reliability was ICC = 0.97 (CI 0.9 - 0.99) and the standard error of measurement was 3.5%.[5] This was considered valid in terms (as a major part of a testing battery) of predicting a subsequent ACL injury. [1, 2, 6]

The Drop jump screening test followed. For this, the participants took a bipedal hip-width stance on a box with a 32 cm target height. A bipedal Drop jump subsequently followed: frontal step – drop – reactive jump, with the shortest possible ground contact time. The normalised knee joint separation as the outcome during the Drop jump was rated at the initial ground contact at the end of the drop from the box and at the lowest point of the body's centre of gravity at the jump’s reversal point. At each of these points, the distances between the hip joints and between the middle of the two knees were measured. The percentage of the knee distance in comparison to the hip distance was calculated using the video analysis software Kinovea (France) to build the normalised knee distance. The normalisation approach allows one to eliminate a potential skewness of the smartphone position by the comparison of the knee separation distance to the hip width (which is in the same plane as the knees). The measurement setup chosen and the subsequent analysis are highly inter-rater reliable (objective, κ = 0.92; 95% confidence interval = 0.829–0.969) and moderately intra-rater reliable (κ = 0.55; 95% confidence interval = 0.49–0.61) [8], respectively. In the same validation study, a sensitivity of 63% and a specificity of 83% for the classification of “high risk” participants (index group identified by expert observers) of the test was found. A normalised knee separation distance at the jump’s reversal point of 60% was selected as the cut-off threshold for the following ratings and analyses [7].

For the sagittal plane landing quality rating, the Balance front hop test [4] was performed. The frontal plane landing quality rating was undertaken using the Balance side hop test [4].. The participants hopped over a square on the floor with a 40x40 cm edge length with their hands on their hips. The end position after landing had to be kept for at least three continuous seconds. Two hops per trial and leg were performed; the better attempt was selected to be further analysed. For both tests, the quality rating criteria at and after landing comprised (1) adequate foot placement, whole sole supported, foot remained stable on the ground after landing, (2a) appropriate medial/lateral position control, knees remained in the sagittal plane, (2b) adequate knee/hip flexion, a sufficient knee flexion was performed, (3a) no lateral trunk motion, and (3b) aligned parallel to the lower leg, no excessive trunk flexion and the trunk remained in the sagittal and transversal planes [4]. Each criterion was rated as successfully performed (1 point) or not (0 point). Sum scores were built side-specific. The two Balance hops show Kappa values of between 0.64 (Balance front hop) and 0.79 (Balance side hop). The percentage of exact agreement between examiners can be excellent (83-93%, depending on the leg and the test); these results are not, as yet, published [3].

**2 Additional figures and tables**


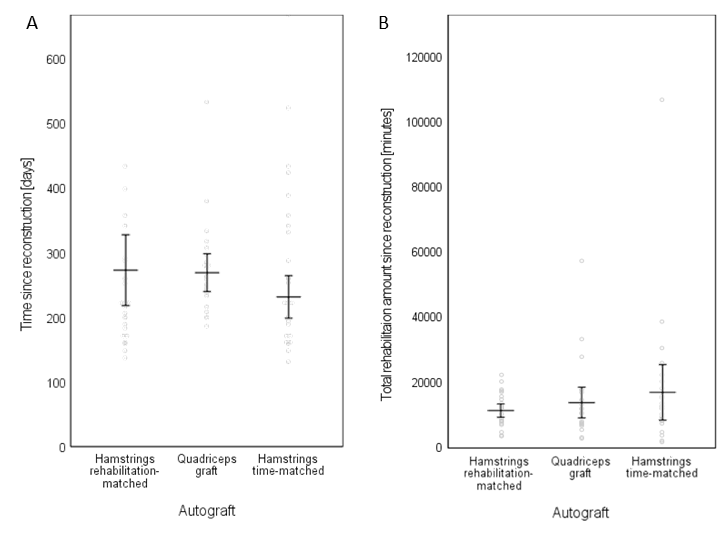


Additional Figure 1: Time since reconstruction (A) and total minutes of rehabilitation since the reconstruction. Data are displayed as individual values (dots) and as group means (horizontal lines) with 95% confidence intervals (vertical lines).

Additional Table 1: Main outcomes of the linear mixed models. For each outcome, the main between-graft effect, potential contributors and the interaction effects of the graft type and each potential contributor are shown as the estimates (above) and the estimates' 95% confidence intervals (in brackets). Part A displays the patients’ reported outcomes, while part B shows the quantitative outcomes and part C the qualitative outcomes.

| - A - Self-reported outcomes | | | | | | |
| --- | --- | --- | --- | --- | --- | --- |
| Estimate | Knee problems ‑ sport (KOOS SPORT) | Knee problems ‑ pain (KOOS PAIN) | Knee problems ‑ symptoms (KOOS SYMTOMS) | Knee problems ‑ everyday function (KOOS ADL) | Confidence to return to sport (ACL‑RSI) | Kinesiophobia (TSK) |
| [95% confidence interval] |  |  |  |  |  |  |
|  |  |  |  |  |  |  |
| Intercept | 89.71 | 115.44 | 70.99 | 137.29 | 15.48 | 23.4 |
|  | [28.68 to 150.74] | [76.72 to 154.15] | [13.12 to 128.86] | [110.09 to 164.49] | [‑49.99 to 80.96] | [8.16 to 38.64] |
| Graft type: hamstring (1) or quadriceps (2) | ‑13.2 | ‑35.47 | ‑9.44 | ‑49.73 | ‑1.48 | 4.1 |
|  | [‑112 to 85.59] | [‑98.14 to 27.2] | [‑103.13 to 84.23] | [‑93.77 to ‑5.7] | [‑107.48 to 104.51] | [‑20.57 to 28.78] |
| Sex/gender (1 = female, 2 = male) | 2 | ‑3.57 | ‑1.9 | ‑0.33 | 10.48 | 1.11 |
|  | [‑12.58 to 16.59] | [‑12.82 to 5.68] | [‑15.74 to 11.92] | [‑6.84 to 6.16] | [‑5.16 to 26.14] | [‑2.52 to 4.76] |
| Graft type*sex | 9.28 | 5.59 | 9.73 | ‑0.12 | ‑2.09 | ‑8.9 |
|  | [‑16.73 to 35.29] | [‑10.9 to 22.09] | [‑14.92 to 34.4] | [‑11.72 to 11.46] | [‑29.99 to 25.81] | [‑15.39 to ‑2.4] |
| Age [years] | ‑1.86 | ‑1.09 | ‑0.5 | ‑0.88 | ‑0.21 | 0.14 |
|  | [‑3.46 to ‑0.26] | [‑2.11 to ‑0.08] | [‑2.01 to 1.01] | [‑1.59 to ‑0.17] | [‑1.92 to 1.5] | [‑0.25 to 0.54] |
| Graft type*age | 1.69 | 0.89 | 0.54 | 1.04 | 0.83 | ‑0.05 |
|  | [‑0.42 to 3.82] | [‑0.45 to 2.23] | [‑1.46 to 2.55] | [0.09 to 1.98] | [‑1.44 to 3.11] | [‑0.58 to 0.47] |
| Body mass index  [kg/m²] | ‑0.26 | 0.07 | ‑0.04 | ‑0.6 | ‑0.02 | ‑0.18 |
|  | [‑1.9 to 1.37] | [‑0.97 to 1.11] | [‑1.6 to 1.51] | [‑1.33 to 0.12] | [‑1.79 to 1.73] | [‑0.59 to 0.22] |
| Graft type*body mass index | ‑0.14 | ‑0.18 | ‑0.6 | 0.23 | ‑1.68 | 0.33 |
|  | [‑3.35 to 3.07] | [‑2.22 to 1.85] | [‑3.64 to 2.44] | [‑1.19 to 1.66] | [‑5.13 to 1.76] | [‑0.46 to 1.13] |
| Time since reconstruction  [days] | 0.03 | 0.02 | 0.06 | 0 | 0.06 | ‑0.02 |
|  | [‑0.03 to 0.09] | [‑0.02 to 0.06] | [0 to 0.13] | [‑0.02 to 0.03] | [‑0.01 to 0.13] | [‑0.04 to ‑0.01] |
| Graft type*time since reconstruction | ‑0.01 | 0 | ‑0.02 | 0 | 0 | 0.02 |
|  | [‑0.13 to 0.1] | [‑0.07 to 0.07] | [‑0.14 to 0.08] | [‑0.05 to 0.05] | [‑0.13 to 0.12] | [0 to 0.05] |
| Time between injury and reconstruction | 0.09 | ‑0.01 | 0.02 | ‑0.02 | 0.03 | 0.02 |
|  | [‑0.01 to 0.2] | [‑0.08 to 0.05] | [‑0.07 to 0.13] | [‑0.07 to 0.01] | [‑0.08 to 0.14] | [0 to 0.05] |
| Graft type*time injury‑reconstruction | ‑0.08 | 0.02 | ‑0.02 | 0.03 | 0 | ‑0.01 |
|  | [‑0.2 to 0.02] | [‑0.04 to 0.09] | [‑0.13 to 0.07] | [‑0.01 to 0.08] | [‑0.12 to 0.11] | [‑0.04 to 0] |
| During (1) or not during (2) lockdown | ‑7.48 | ‑11.43 | ‑13.1 | ‑7.72 | ‑13.2 | 7.51 |
|  | [‑22.74 to 7.76] | [‑21.11 to ‑1.76] | [‑27.56 to 1.35] | [‑14.52 to ‑0.92] | [‑29.57 to 3.15] | [3.7 to 11.32] |
| Graft type*lockdown | 11.27 | 13.3 | 19.85 | 11.29 | 24.47 | ‑6.96 |
|  | [‑11.23 to 33.79] | [‑0.97 to 27.58] | [‑1.49 to 41.2] | [1.25 to 21.33] | [0.31 to 48.63] | [‑12.59 to ‑1.34] |
| Tegner activity scale | 3.1 | 2 | 4.02 | 1.15 | 4.03 | ‑1.27 |
|  | [‑0.53 to 6.73] | [‑0.29 to 4.31] | [0.58 to 7.47] | [‑0.46 to 2.77] | [0.13 to 7.92] | [‑2.18 to ‑0.36] |
| Graft type*Tegner activity scale | ‑5.35 | ‑0.9 | ‑4.21 | ‑0.49 | ‑2.14 | 2.02 |
|  | [‑12.14 to 1.43] | [‑5.21 to 3.4] | [‑10.65 to 2.22] | [‑3.52 to 2.53] | [‑9.43 to 5.13] | [0.32 to 3.71] |
| Rehabilitation/training frequency  [week^‑1^] | 1.95 | 0.7 | ‑0.94 | ‑0.37 | 3.64 | ‑0.23 |
|  | [‑2.14 to 6.05] | [‑1.89 to 3.3] | [‑4.83 to 2.94] | [‑2.2 to 1.45] | [‑0.75 to 8.04] | [‑1.26 to 0.78] |
| Graft type*frequency | ‑2.21 | ‑2.01 | 0.18 | 0.64 | ‑1.67 | ‑0.74 |
|  | [‑8.06 to 3.64] | [‑5.73 to 1.69] | [‑5.36 to 5.73] | [‑1.96 to 3.25] | [‑7.95 to 4.6] | [‑2.2 to 0.71] |
| - B - Front hop for distance and drop jump | | | | | | |
| Estimate | Front hop for distance ACL leg | Front hop for distance contralateral leg | Front hop for distance limb symmetry index | Normalised knee separation distance at landing | Normalised knee separation distance at lowest point | Normalised knee separation distance transition |
| [95% confidence interval] |  |  |  |  |  |  |
| Intercept | 112.27 | 121.38 | 0.88 | 81.78 | 78.8 | ‑2.97 |
|  | [10.93 to 213.6] | [33.04 to 209.72] | [0.38 to 1.38] | [15.87 to 147.69] | [‑10.43 to 168.05] | [‑53.02 to 47.07] |
| Graft type: hamstrings (1) or quadriceps (2) | ‑52.82 | ‑42.98 | 0 | ‑22.77 | ‑10.82 | ‑7.23 |
|  | [‑216.87 to 111.21] | [‑186 to 100.03] | [‑0.81 to 0.8] | [‑129.47 to 83.93] | [‑155.3 to 133.64] | [‑88.26 to 73.78] |
| Sex/gender (1 = female, 2 = male) | 15.99 | 15.69 | 0.02 | 4.3 | 4.38 | 0.07 |
|  | [‑8.22 to 40.22] | [‑5.42 to 36.81] | [‑0.09 to 0.14] | [‑11.45 to 20.06] | [‑16.95 to 25.72] | [‑11.88 to 12.04] |
| Graft type*sex | 3.96 | ‑0.28 | 0.12 | ‑6.74 | ‑17.43 | ‑2.64 |
|  | [‑39.22 to 47.15] | [‑37.94 to 37.36] | [‑0.09 to 0.33] | [‑34.84 to 21.34] | [‑55.47 to 20.59] | [‑23.97 to 18.68] |
| Age  [years] | ‑0.15 | 0.37 | 0 | ‑0.92 | ‑0.86 | 0.06 |
|  | [‑2.81 to 2.49] | [‑1.93 to 2.69] | [‑0.01 to 0] | [‑2.65 to 0.79] | [‑3.2 to 1.47] | [‑1.24 to 1.37] |
| Graft type*age | ‑0.47 | ‑0.33 | ‑0.004 | 2.37 | 1.51 | ‑0.53 |
|  | [‑4 to 3.04] | [‑3.41 to 2.73] | [‑0.02 to 0.01] | [0.08 to 4.67] | [‑1.59 to 4.62] | [‑2.27 to 1.2] |
| Body mass index  [kg/m²] | ‑1.72 | ‑1.47 | ‑0.003 | 0.53 | 0.94 | 0.4 |
|  | [‑4.44 to 1] | [‑3.84 to 0.9] | [‑0.01 to 0.01] | [‑1.23 to 2.31] | [‑1.45 to 3.34] | [‑0.94 to 1.74] |
| Graft type*body mass index | 0.49 | ‑0.21 | 0.001 | ‑1.26 | 1.83 | 2.58 |
|  | [‑4.83 to 5.82] | [‑4.86 to 4.43] | [‑0.02 to 0.02] | [‑4.73 to 2.2] | [‑2.86 to 6.53] | [‑0.04 to 5.22] |
| Additional Table continued | | | | | | |
|  |  |  |  |  |  |  |
| Time since reconstruction  [days] | ‑0.0017 | ‑0.05 | 0.0004 | 0.05 | 0.02 | ‑0.02 |
|  | [‑0.11 to 0.11] | [‑0.15 to 0.04] | [‑0.0001 to 0.001] | [‑0.02 to 0.12] | [‑0.07 to 0.12] | [‑0.07 to 0.03] |
| Graft type*time since reconstruction | 0.07 | 0.1 | ‑0.0006 | ‑0.08 | ‑0.13 | ‑0.04 |
|  | [‑0.12 to 0.27] | [‑0.07 to 0.28] | [‑0.0016 to 0.0003] | [‑0.21 to 0.04] | [‑0.31 to 0.04] | [‑0.14 to 0.05] |
| Time between injury and reconstruction | 0.02 | ‑0.02 | 0.0005 | ‑0.01 | 0.05 | 0.07 |
|  | [‑0.15 to 0.2] | [‑0.18 to 0.12] | [‑0.0003 to 0.0014] | [‑0.12 to 0.1] | [‑0.09 to 0.21] | [‑0.01 to 0.16] |
| Graft type*time injury‑reconstruction | ‑0.04 | 0 | ‑0.0004 | 0.02 | ‑0.05 | ‑0.08 |
|  | [‑0.23 to 0.14] | [‑0.16 to 0.16] | [‑0.0014 to 0.0004] | [‑0.09 to 0.14] | [‑0.21 to 0.11] | [‑0.17 to 0.01] |
| During (1) or not during (2) lockdown | ‑14.04 | ‑2.22 | ‑0.1 | ‑10.34 | ‑7.06 | 3.28 |
|  | [‑39.37 to 11.27] | [‑24.29 to 19.85] | [‑0.22 to 0.02] | [‑26.82 to 6.12] | [‑29.36 to 15.23] | [‑9.22 to 15.79] |
| Graft type*lockdown | 10.79 | ‑13.75 | 0.23 | 15.46 | 8.35 | ‑7.23 |
|  | [‑26.59 to 48.17] | [‑46.34 to 18.83] | [0.05 to 0.42] | [‑8.84 to 39.78] | [‑24.57 to 41.27] | [‑25.69 to 11.23] |
| Tegner activity scale | 3.4 | 1.69 | 0.02 | 2.65 | 0.37 | ‑2.27 |
|  | [‑2.63 to 9.43] | [‑3.56 to 6.96] | [0 to 0.05] | [‑1.27 to 6.57] | [‑4.94 to 5.68] | [‑5.26 to 0.7] |
| Graft type*Tegner activity scale | ‑0.19 | 5.42 | ‑0.04 | 2.01 | 1.21 | ‑0.1 |
|  | [‑11.47 to 11.08] | [‑4.4 to 15.26] | [‑0.09 to 0.01] | [‑5.31 to 9.35] | [‑8.71 to 11.14] | [‑5.68 to 5.46] |
| Rehabilitation/training frequency  [week^‑1^] | 1.62 | ‑0.4 | 0.01 | 0.24 | ‑0.79 | ‑1.03 |
|  | [‑5.17 to 8.43] | [‑6.34 to 5.52] | [‑0.01 to 0.05] | [‑4.18 to 4.67] | [‑6.79 to 5.19] | [‑4.4 to 2.32] |
| Graft type* frequency | 1.36 | 3.11 | 0 | ‑3.29 | ‑4.53 | 0.83 |
|  | [‑8.36 to 11.08] | [‑5.35 to 11.59] | [‑0.05 to 0.03] | [‑9.61 to 3.03] | [‑13.09 to 4.03] | [‑3.96 to 5.63] |
| - C - Balance hops | | | | | | |
| Estimate | Balance side hop ‑ ACL leg | Balance side hop Contralateral leg | Balance front hop ‑ between‑leg difference | Balance front hop ‑ ACL leg | Balance front hop Contralateral leg | Balance front hop ‑ between‑leg difference |
| [95% confidence interval] |  |  |  |  |  |  |
| Intercept | 3.65 | 6.66 | 0.4 | 3.82 | 4.3 | 0.85 |
|  | [‑0.21 to 7.53] | [2.33 to 10.99] | [‑3.35 to 4.17] | [‑0.5 to 8.15] | [1.33 to 7.26] | [‑3.38 to 5.09] |
| Graft type: hamstrings (1) or quadriceps (2) | ‑3.1 | ‑6.87 | 1 | ‑0.27 | ‑3.66 | ‑0.02 |
|  | [‑9.37 to 3.16] | [‑13.88 to 0.14] | [‑5.08 to 7.09] | [‑7.28 to 6.74] | [‑8.46 to 1.13] | [‑6.89 to 6.83] |
| Sex/gender (1 = female, 2 = male) | 0.2 | 0.81 | ‑0.22 | 0.31 | 0.1 | 0.15 |
|  | [‑0.72 to 1.12] | [‑0.21 to 1.85] | [‑1.12 to 0.67] | [‑0.71 to 1.35] | [‑0.6 to 0.8] | [‑0.85 to 1.17] |
| Graft type*sex | 0.27 | ‑1.55 | 0.74 | ‑0.08 | 0.21 | ‑0.22 |
|  | [‑1.37 to 1.93] | [‑3.4 to 0.29] | [‑0.85 to 2.35] | [‑1.93 to 1.75] | [‑1.05 to 1.47] | [‑2.02 to 1.58] |
| Age [years] | 0.01 | 0 | 0 | ‑0.04 | 0 | 0.03 |
|  | [‑0.09 to 0.11] | [‑0.1 to 0.11] | [‑0.08 to 0.1] | [‑0.15 to 0.06] | [‑0.07 to 0.08] | [‑0.07 to 0.14] |
| Graft type*age | 0.03 | 0.02 | 0 | 0.01 | 0.06 | ‑0.01 |
|  | [‑0.09 to 0.17] | [‑0.12 to 0.17] | [‑0.13 to 0.12] | [‑0.13 to 0.17] | [‑0.03 to 0.17] | [‑0.16 to 0.13] |
| Body mass index [kg/m²] | ‑0.04 | ‑0.16 | 0.01 | 0 | ‑0.03 | ‑0.04 |
|  | [‑0.15 to 0.05] | [‑0.28 to ‑0.04] | [‑0.08 to 0.12] | [‑0.12 to 0.11] | [‑0.11 to 0.04] | [‑0.15 to 0.07] |
| Graft type*body mass index | ‑0.02 | 0.11 | ‑0.02 | ‑0.08 | 0.07 | 0.02 |
|  | [‑0.22 to 0.17] | [‑0.11 to 0.34] | [‑0.22 to 0.16] | [‑0.31 to 0.14] | [‑0.08 to 0.22] | [‑0.2 to 0.24] |
| Time since reconstruction  [days] | 0.00008 | ‑0.00107 | 0.00029 | ‑0.00036 | 0.00046 | ‑0.00028 |
|  | [‑0.004 to 0.004] | [‑0.006to 0.004] | [‑0.004 to 0.005] | [‑0.005 to 0.005] | [‑0.003 to 0.004] | [‑0.005 to 0.004] |
| Graft type*time since reconstruction | 0.00615 | 0.00735 | ‑0.0014 | 0.0046 | 0.00315 | 0.00398 |
|  | [‑0.001 to 0.01] | [‑0.001 to 0.02] | [‑0.009to 0.006] | [‑0.004 to 0.01] | [‑0.003 to 0.009] | [‑0.004 to 0.01] |
| Time between injury and reconstruction | 0.00189 | ‑0.00019 | 0.00486 | ‑0.00208 | 0.00004 | 0.00438 |
|  | [‑0.005 to 0.009] | [‑0.008 to 0.007] | [‑0.002 to 0.01] | [‑0.01 to 0.006] | [‑0.005 to 0.005] | [‑0.003 to 0.01] |
| Graft type*time injury‑reconstruction | ‑0.00346 | 0.00012 | ‑0.00595 | 0.00274 | 0.00117 | ‑0.00527 |
|  | [‑0.01 to 0.003] | [‑0.008 to 0.008] | [‑0.01 to 0.001] | [‑0.005to 0.01] | [‑0.004 to 0.007] | [‑0.01 to 0.003] |
|  |  |  |  |  |  |  |
| - C - Balance hops (continued) | | | | | | |
| Estimate | Balance side hop ‑ ACL leg | Balance side hop Contralateral leg | Balance front hop ‑ between‑leg difference | Balance front hop ‑ ACL leg | Balance front hop Contralateral leg | Balance front hop ‑ between‑leg difference |
| [95% confidence interval] |  |  |  |  |  |  |
|  | [‑1.41 to 0.51] | [‑0.97 to 1.19] | [‑0.41 to 1.46] | [‑0.79 to 1.37] | [‑0.12 to 1.36] | [‑0.96 to 1.15] |
| Graft type*lockdown | 0.86 | 1.09 | ‑0.89 | 0.32 | ‑0.72 | 0.07 |
|  | [‑0.56 to 2.29] | [‑0.49 to 2.69] | [‑2.28 to 0.49] | [‑1.27 to 1.92] | [‑1.82 to 0.36] | [‑1.48 to 1.64] |
| Tegner activity scale | 0.15 | ‑0.13 | ‑0.16 | ‑0.02 | ‑0.04 | ‑0.09 |
|  | [‑0.07 to 0.38] | [‑0.39 to 0.12] | [‑0.38 to 0.06] | [‑0.28 to 0.23] | [‑0.21 to 0.13] | [‑0.34 to 0.15] |
| Graft type*Tegner activity scale | 0.06 | 0.5 | 0.12 | 0.02 | ‑0.24 | 0.1 |
|  | [‑0.36 to 0.49] | [0.02 to 0.98] | [‑0.28 to 0.54] | [‑0.45 to 0.5] | [‑0.57 to 0.08] | [‑0.36 to 0.57] |
| Rehabilitation/training frequency  [week^—1^] | 0.14 | 0.22 | 0 | 0.18 | ‑0.04 | 0.08 |
|  | [‑0.11 to 0.4] | [‑0.06 to 0.51] | [‑0.24 to 0.26] | [‑0.1 to 0.47] | [‑0.24 to 0.15] | [‑0.2 to 0.36] |
| Graft type* frequency | ‑0.16 | ‑0.21 | ‑0.04 | ‑0.03 | 0.28 | ‑0.22 |
|  | [‑0.53 to 0.21] | [‑0.62 to 0.2] | [‑0.4 to 0.31] | [‑0.45 to 0.37] | [0 to 0.56] | [‑0.63 to 0.18] |

KOOS, Knee Injury and Osteoarthritis Outcome Score; SPORT, sport sub-scale; PAIN, pain sub-scale; SYMPTOMS, symptom sub-scale; ADL, activities of daily living sub-scale; ACL-RSI, return to sport after injury; TSK, Tampa scale of kinesiophobia.

3 S**upplemental** References

1. Ashigbi EYK, Banzer W, Niederer D (2020) Return to Sport Tests' Prognostic Value for Reinjury Risk after Anterior Cruciate Ligament Reconstruction: A Systematic Review. Med Sci Sports Exerc 52:1263–1271. doi: 10.1249/MSS.0000000000002246

2. Grindem H, Snyder-Mackler L, Moksnes H et al. (2016) Simple decision rules can reduce reinjury risk by 84% after ACL reconstruction: the Delaware-Oslo ACL cohort study. Br J Sports Med 50:804–808. doi: 10.1136/bjsports-2016-096031

3. Keller M, Niederer D, Schwesig R et al. Lower extremity movement quality in professional team sport athletes: Inter-rater reliability and relationships with quantitative results from the corresponding pattern. unpublished

4. Keller M, Kurz E, Schmidtlein O et al. (2016) Interdisciplinary Assessment Criteria for Rehabilitation after Injuries of the Lower Extremity: A Function-Based Return to Activity Algorithm. (Interdisciplinary Assessment Criteria for Rehabilitation after Injuries of the Lower Extremity: A Function-Based Return to Activity Algorithm). Sportverletzung Sportschaden 30:38–49. doi: 10.1055/s-0042-100966

5. Kockum B, Heijne AI-LM (2015) Hop performance and leg muscle power in athletes: Reliability of a test battery. Phys Ther Sport 16:222–227. doi: 10.1016/j.ptsp.2014.09.002

6. Kyritsis P, Bahr R, Landreau P et al. (2016) Likelihood of ACL graft rupture. Not meeting six clinical discharge criteria before return to sport is associated with a four times greater risk of rupture. Br J Sports Med 50:946–951. doi: 10.1136/bjsports-2015-095908

7. Noyes FR, Barber-Westin SD, Fleckenstein C et al. (2005) The drop-jump screening test: difference in lower limb control by gender and effect of neuromuscular training in female athletes. Am J Sports Med 33:197–207. doi: 10.1177/0363546504266484

8. Redler LH, Watling JP, Dennis ER et al. (2016) Reliability of a field-based drop vertical jump screening test for ACL injury risk assessment. Phys Sportsmed 44:46–52. doi: 10.1080/00913847.2016.1131107
